# Increased Cardiometabolic Risk in Dynapenic Obesity: Results from the Study of Workers’ Health (ESAT)

**DOI:** 10.3390/life14091174

**Published:** 2024-09-18

**Authors:** Mariana de Oliveira Carvalho, Alice Pereira Duque, Grazielle Vilas Bôas Huguenin, Mauro Felippe Felix Mediano, Luiz Fernando Rodrigues Júnior

**Affiliations:** 1 Education and Research Department, National Institute of Cardiology, Rio de Janeiro 22240-002, RJ, Brazil; mariana.olicarvalho@gmail.com (M.d.O.C.); alicepereiraduque@gmail.com (A.P.D.); graziellevbhuguenin@gmail.com (G.V.B.H.); mffmediano@gmail.com (M.F.F.M.); 2 Nutrition and Dietetics Department, Fluminense Federal University, Niterói 24020-140, RJ, Brazil; 3 Evandro Chagas National Institute of Infectious Diseases, Oswaldo Cruz Foundation, Rio de Janeiro 21040-900, RJ, Brazil; 4 Department of Physiological Sciences, Federal University of the State of Rio de Janeiro, Rio de Janeiro 20211-010, RJ, Brazil

**Keywords:** dynapenic obesity, dynapenic abdominal obesity, cardiovascular risk, cardiometabolic risk

## Abstract

**Background:** The coexistence of obesity and low muscle strength—denoted dynapenic obesity (DO)—has been associated with an unhealthy metabolic profile and increased risk for metabolic syndrome. However, there is a lack on studies investigating if DO exhibits higher cardiometabolic risk than non-dynapenic obesity. **Objectives:** To assess if individuals with DO exhibit elevated cardiometabolic risk compared to non-dynapenic obesity. **Methods:** a cross-sectional study that analyzed the data of workers from a quaternary care hospital collected between November 2018 and March 2020. Participants were stratified into the following anthropometrical and peripheral muscle strength profiles: non-obese/non-dynapenic (NOND), non-obese/dynapenic (NOD), obese/non-dynapenic (OND), and obese dynapenic (OD). Cardiovascular risk was evaluated by Atherogenic Index (AI), Plasma Atherogenic Index (PAI), Hypertriglyceridemic Waist (HW), A Body Shape Index (ABSI), Atherogenic Dyslipidemia (AD), Castelli Indices I and II, and Framingham Score (FS). **Results:** the OD group had significantly lower HDL compared to all others (*p* = 0.009), and despite exhibited lower prevalence of HW compared to OND (*p* < 0.01), a higher cardiometabolic risk compared to OND profile was observed assessing AI (*p* = 0.05), Castelli I (*p* < 0.05) and Castelli II (*p* < 0.05) scores. **Conclusions:** in the studied population, individuals with DO exhibit elevated cardiometabolic risk compared to other anthropometrical and peripheral muscle strength profiles.

## 1. Introduction

According to the World Health Organization (WHO), approximately one billion people in the world presented with obesity in 2022 [1]. The worldwide prevalence of obesity has substantially increased over the last 40 years, from 3% to 11% in men and from 6% to 15% in women, posing a significant global public health challenge associated with a higher risk of cardiovascular diseases and mortality [2,3].

Therefore, the assessment of cardiovascular risk, either using specific parameters such as lipid profile, blood glucose, comorbidities and lifestyle, or by calculating prediction scores that combine specific parameters such as the Framingham score, can be relevant for the prevention of cardiovascular events occurrence [4,5]. Nevertheless, mainly in the context of primary care, the costs related to the laboratorial screening of all the individuals are a potential barrier for cardiovascular risk stratification [6]. In this context, the identification of higher cardiovascular risk in individuals with specific of anthropometrical and functional factors—as muscle strength—could serve as an initial strategy for identification of individuals exposed to higher cardiovascular risk for later more expensive screenings, allowing a better resources allocation.

The concomitant lifestyle changes, such as a physically inactive lifestyle and inadequate nutrition, associated with a higher occurrence of chronic diseases (such as diabetes and obesity), may affect muscle function with decrease in skeletal muscle strength, a condition called dynapenia [7,8,9]. Initially associated to the aging process, 10% of adults from 50 to 59 years old, and 23% of patients with chronic diseases present dynapenia, with its prevalence varying depending on the diagnostic criteria applied [9].

In recent decades, the coexistence of excess body fat and low muscle strength—which defines dynapenic obesity (DO)—has been associated with deleterious metabolic profile, increased risk of metabolic syndrome and higher cardiovascular mortality [6,7,8]. However, due to the heterogeneity of populations and diagnostic methods employed to diagnose DO, studies investigating the association between DO and adverse clinical outcomes, especially focusing on cardiovascular risk, are scarce [10,11]. So, the present study aims to assess if individuals with DO exhibit elevated cardiometabolic risk compared to non-dynapenic.

## 2. Materials and Methods

### 2.1. Study Design and Participants

The present study is associated with a broader observational study entitled “Evaluation of stress indicators, body composition, and metabolic profile in employees of a cardiology reference hospital: contributions to quality of life—the Worker Health Study (ESAT)”, conducted at the National Institute of Cardiology (NIC), from November, 2018 to March, 2020. The inclusion criteria of ESAT study comprised: being active employees of NIC and being aged ≥ 18 years. The exclusion criteria were: being on medical sick leave, being assigned to another healthcare unit, having undergone recent surgery, fasting for more than 13 h, being pregnant and/or lactating, not responding to the team’s attempts to contact for data collection (minimum of three attempts), or undergoing the second day of collection (D2) after more than two months from the first day of collection (D1). The ESAT study resulted in a comprehensive database, used in the present study, as a convenient sample size [12]. For the present study, we also excluded participants with body mass index (BMI) ≤ 18.5 kg/m^2^ or those with missing information for the characterization of the anthropometric or peripheral muscle strength profiles.

The present study followed the recommendations of STROBE (Strengthening the Reporting of Observational Studies in Epidemiology), approved by ethics committee of the National Institute of Cardiology (CAAE: 96222718.7.0000.5272/Opinion: 5.046.117).

### 2.2. Anthropometric Measurement

Anthropometric data consisted of weight, height, BMI calculation, waist circumference (WC, in cm), and hip circumference (HC, in cm) measurements, according to WHO recommendations and with tools and procedures better described by Araújo et al. [1,12]. The BMI was calculated as weight (kg) divided by height squared (m^2^) and classified as non-obese (18.5–29.9 kg/m^2^) or obesity (≥30 kg/m^2^) [12].

### 2.3. Comorbidities

Comorbidities were obtained from an ESAT study database as referred by volunteers, and consisted of hypertension, dyslipidemia, hypothyroidism, and smoking (categorized in non-smoker, smoker or former smoker).

### 2.4. Physical Activity Level Assessment

The short version of the International Physical Activity Questionnaire (IPAQ-SF), already tested for validity and reliability [13,14], was used to evaluate physical activity levels. The IPAQ-SF consists of 7 questions assessing the frequency, time and intensity of physical activity performed at work, leisure time, commuting and household, and the time seated on a typical week and weekend day, allowing individuals to be classified into three different categories: high, moderate and low physical activity levels [15,16].

### 2.5. Peripheral Muscle Strength

The handgrip strength of dominant hand was used as a proxy of peripheral muscle strength. Dynapenia classification was based on the 30th percentile of grip strength of the population in a study conducted in 2008 by Shülussel et al., which presented reference values for dynamometry for healthy Brazilian adults aged 20 and older, stratified by age group and sex [17]. Individuals were classified as non-dynapenic (handgrip above cutoff point) or dynapenic (handgrip below cutoff point).

### 2.6. Characterization of Anthropometrical and Peripheral Muscle Strength Profile

The profiles based on anthropometric and peripheral muscle strength were as follows:

- Non-obese/Non-dynapenic (NOND): individuals with BMI < 30 kg/m^2^ and handgrip strength within the predicted range for their sex and age.
- Non-obese/Dynapenic (NOD): individuals with BMI < 30 kg/m^2^ and handgrip strength below the predicted range for their sex and age.
- Obese/Non-dynapenic (OND): individuals with BMI ≥ 30 kg/m^2^ but with preserved handgrip strength.
- Obese/dynapenic (OD): individuals with both obesity assessed by BMI ≥ 30 kg/m^2^ and low handgrip strength verified by dynamometry values below the predicted range for their sex and age.

### 2.7. Cardiovascular Risk Assessment

Cardiovascular risk was assessed using fasting glucose, lipid profile (total cholesterol; high-density lipoprotein [HDL]; calculated low-density lipoprotein [LDL], and triglycerides [TG]), and by the following cardiovascular risk scores: Atherogenic Index (AI) [18,19]; Plasma Atherogenic Index (PAI) [18,20], Hypertriglyceridemic Waist (HW) [21], A Body Shape Index (ABSI) [22], Atherogenic Dyslipidemia (AD) [23], Castelli Index I and II [5], and Framingham Score (FS) [4,24] (Table 1).

AI is a predictor of cardiovascular risk with easy applicability and reproducibility and easy calculation from a simple lipid profile. It is calculated as the ratio of non-HDL cholesterol to HDL cholesterol using the formula described in Table 1. AI values < 2 are considered low cardiovascular risk [18,19].

PAI uses the relationship between plasma TG concentration and non-HDL cholesterol (HDL-c) calculated using the formula described in Table 1. PAI shows a positive association with cardiovascular risk and its secondary outcomes, classified into low risk (PAI < 0.11), medium risk (PAI between 0.11 and 0.21), and increased risk (>0.21) [18,20].

Hypertriglyceridemic Waist (HW) is the condition in which the individual simultaneously presents increased waist circumference and hypertriglyceridemia. In this study, the HW phenotype was considered present when the volunteer simultaneously presented WC ≥ 88 cm in women and ≥102 cm in men, and TG ≥ 150 mg/dL, according to the National Cholesterol Education Program (NCEP) [21].

ABSI adjusts waist circumference for weight and height, such that ABSI indicates a waist circumference greater than predicted by weight and height, suggesting increased body concentration mainly in the trunk. With this adjustment, it is possible to evaluate overweight and obesity in a less limited manner than BMI. ABSI formula is presented in Table 1 [22].

Atherogenic Dyslipidemia refers to the lipid profile composed of increased serum levels of TG, normal or slightly increased levels of LDL cholesterol with molecular reduction of the same, and low levels of HDL cholesterol simultaneously. This phenotype is associated with metabolic syndrome, already known as a cardiovascular risk factor [25]. The reference values will be TG ≥ 150 mg/dL with HDL < 40 mg/dL with LDL > 130 mg/dL for men; TG ≥ 150 mg/dL with HDL < 50 mg/dL and LDL > 130 mg/dL for women and/or TG ≥ 150 mg/dL with HDL < 40 mg/dL (all) with LDL > 130 mg/dL for both sexes in case of borderline values for sexes or lack of information on some variables [23].

Castelli Index I and Castelli Index II (30, 31) have been able to identify individuals at risk of for atherosclerosis cardiovascular events and assist in the evaluation of lifestyle intervention effects, part of good practices in cardiovascular disease prevention [26,27]. Their normality values are Castelli Index I > 4.4 in women and >5.1 in men and Castelli Index II > 2.9 in women and >3.3 in men, respectively [5].

The Framingham Score is calculated using information on age, total cholesterol, high-density lipoprotein, systolic blood pressure and its pharmacological management, smoking, and diabetes mellitus (DM). A percentage value of cardiovascular risk chances is assigned to the final sum of these points, and then the individual is classified by risk category, categorized as low risk (<5%), moderate (5–19%), or high (≥20%) [4,24].

### 2.8. Data Analysis

Results are presented as mean ± standard deviation or median and interquartile range. Comparison between groups for parameters with normal distribution (assessed by Shapiro–Wilk normality test) was performed with one-way ANOVA, and for those without normal distribution, using the Kruskal–Wallis tests, followed by post hoc Dunn’s test. The association of categorical variables was assessed by Fisher’s Exact Test. All analyses were performed using the statistics and data science software, STATA 16 (StataCorp, College Station, TX, USA). The *p* < 0.05 was considered statistically significant.

## 3. Results

**Figure 1:**
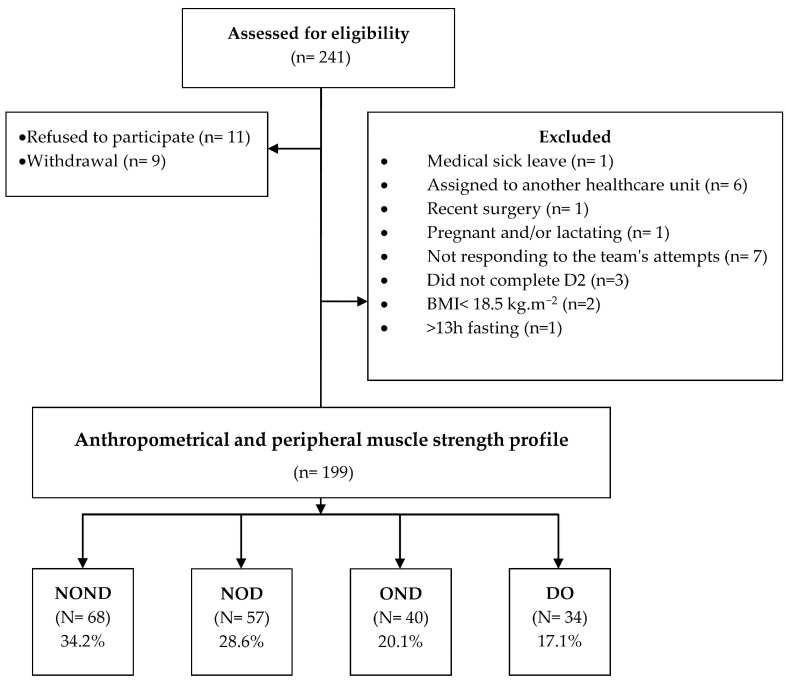
Study’s flowchart. NOND = Non-obese/Non-dynapenic; NOD = Non-obese/Dynapenic; OND = Obese/Dynapenic; OD = Obese Dynapenic.

Of the 241 participants recruited for the study, 199 met inclusion criteria, were included in the study, and classified according to anthropometric and muscle strength profiles into NOND (*n* = 68); NOD (*n* = 57); OND (*n* = 40); and OD (*n* = 34), evidencing the prevalence of DO of 17.1% (Figure 1).

Table 2 presents the descriptive data of the participants of the study. The mean age was 45.1 years, with the majority being women. The major reported comorbidity was hypertension, followed by diabetes, hypothyroidism, and dyslipidemia. Additionally, 17.6% of volunteers were using antihypertensive medications, 5% were using hypoglycemic agents, and 3.5% were using lipid-lowering drugs.

Also, the majority of participants were non-smokers. The median BMI was 28.2 kg/m^2^, and only 26.1% of the sample was categorized as normal weight based on BMI, while the prevalence of obesity was 37.2%. The median waist circumference was 92.0 cm. The median handgrip strength was 40 kgf for men and 23 kgf for women, with almost half of population being dynapenic. The majority of volunteers exhibited moderate/high IPAQ-SF.

Increased cardiometabolic risk according to PAI was observed in majority of the participants, and the phenotypes of hypertriglyceridemic waist and atherogenic dyslipidemia were 13.1% and 5.5%, respectively. The Castelli I Index suggested cardiometabolic risk in approximately quarter of the participants, and the Castelli II Index in a third of them. Regarding cardiovascular risk calculated through the Framingham Score, 43.7% were in the low-risk category, 43.7% in the moderate-risk category, and 12.6% in the increased cardiovascular risk category.

Table 3 presents the comparison of data between the anthropometric and muscle strength profiles. There was no difference in age, sex, height and IPAQ-SF METS or categories between the profiles. Also, there was no difference in mean weight, waist circumference, and BMI between obese profiles (DO vs. NDO), and between non-obese profiles (NOND vs. NOD). The dynapenic profiles (NOD and OD) exhibited lower (*p* < 0.001) handgrip strength compared to non-dynapenic profiles (NOND and OND), but there was no difference in handgrip strength between non-dynapenic (NOND vs. OND) or dynapenic profiles (NOD vs. OD). Regarding comorbidities, the prevalence of hypertension, diabetes and hypothyroidism did not vary between profiles, but dyslipidemia was only reported by OND (*p* = 0.012). Also, the obese profiles exhibited higher use of diuretics compared to non-obese profiles (*p* = 0.018), with no differences in other medications usage between profiles. However, there was no difference in IPAQ-SF between anthropometric and muscle strength profiles. The glycemia, total cholesterol, and LDL did not vary between profiles. However, the DO group exhibited significantly lower HDL compared to all other profiles (0.009). Triglyceride values were higher in obese (OND and DO) compared to non-obese (NOND and NOD) profiles (0.002). The obese profiles (OND and OD) presented higher cardiometabolic risk scores compared to non-obese profiles (NOND and NOD) evidenced by higher AIP (*p* < 0.001), higher prevalence of individuals in IAP high risk category (*p* < 0.002), hypertriglyceridemic waist (*p* < 0.001), Castelli I (*p* < 0.001) and Castelli II (*p* < 0.001) scores. Furthermore, despite DO exhibiting lower prevalence of hypertriglyceridemic waist compared to OND (*p* < 0.01), a higher cardiometabolic risk compared to OND profile was observed assessing AI (*p* = 0.05), Castelli I (*p* < 0.05) and Castelli II (*p* < 0.05) scores. Finally, there was no difference on mean Framingham score, and on prevalence of Castelli, Castelli II or Framingham risk categories.

## 4. Discussion

This was the first study to evaluate the cardiometabolic risk in DO considering a wide variety of risk scores. Our main result was that individuals with DO beyond being exposed to higher cardiometabolic risk compared to non-obese individuals, presented higher cardiometabolic risk compared to those also with obesity but without dynapenia, evidenced by the lowest HDL cholesterol values and higher AI, Castelli I and II scores compared with NOND, NOD, and OND.

The predictive scores for increased cardiometabolic risk uses were based on components of participants’ lipid profile: AIP, hypertriglyceridemic waist, atherogenic dyslipidemia, Castelli Indexes I and II, corroborating with the known relationship between dyslipidemia and increased cardiovascular risk, metabolic syndrome, and type 2 diabetes [5,28]. According to our results, DO seems to augment the atherosclerosis-related indexes (AI, Castelli Indexes I and II), but exerted no impact on general cardiovascular risk (Framingham score) between the anthropometric and muscle strength profiles. Oppositely, a study with 833 individuals evidenced an association between DO and metabolic and lipid profile disorders, notably hypertriglyceridemia, as well as risk factors for metabolic syndrome [29], however, the population of this study was older (around 70 years old) than observed in our study (around 45 years old), thus limiting comparisons between studies, since HDL was the only lipid profile variable worse in DO compared to OND. So, maybe, the predictive scores of cardiometabolic risk could better reflect the relationship between lipid profile and increased cardiometabolic risk [30,31].

Regarding the general population of our study, it draws attention that despite the low age, the majority of the population exhibited intermediate/high risk according to the AIP and Framingham score, even with the majority of volunteers exhibiting moderate/high physical activity levels. Also, the results revealed a high prevalence of obesity in the sample, with 37.2% of participants categorized as obese. Although this rate is nearly three times higher than the most recent WHO data on the global prevalence of obesity, it seems to be in line with projections of increased obesity prevalence in the population in the coming years [2,3]. When compared to epidemiological data from Brazil, the prevalence of obesity in our study was higher than that reported by the latest Vigitel survey [25]. A study by Moreira et al. that assessed the prevalence of obesity and cardiovascular risk using the Framingham score in the Brazilian population showed overweight and obesity prevalence of 61.9% and increased cardiovascular risk prevalence of 18.9% [32]. For comparison, our overweight and obesity prevalence was 73.9%, relatively higher, although the prevalence of high cardiovascular risk was lower than that in the study.

Interestingly, we found higher prevalence of dynapenia compared to European adults aging from 18 to 74 years, in whose dynapenia ranges from 7.9% in general population [9] to 22.5% in the elderly. In the American elderly population, dynapenia stands at 38.2% [30] while in adults aged 50 years and older in 17.2%, increasing to 28.2% among those aged ≥ 75 years old [33]. The observed discrepancy in our results from the aforementioned studies could be related to difference in cutoff points for each ethnic population, reason why we opted for use the classification based on the 30th percentile of handgrip strength of the population in a study conducted in 2008 by Shülussel et al., which presented reference values for dynamometry for healthy Brazilian adults aged 20 and older, stratified by age group and sex [17]. Also, the occurrence of chronic comorbidities already evidenced as deleterious for muscle mass and strength, such as diabetes, hypertension, low physical activity and smoking was not different comparing DO and other profiles, making emerge a hypothesis of genetic predisposal for this dynapenia beyond the influence of chronic comorbidities [26].

The prevalence of DO was relatively high in our study (17%). A study with 382 Brazilians aged 60 years and older found a prevalence of 10.7% [27]. Our classification for DO was based on BMI and handgrip strength, while the aforementioned study used abdominal obesity, assessed by waist circumference and handgrip strength, which may explain the differences in prevalence of DO between the studies.

An important limitation of the present study was that it was an observational cross-sectional study, which limits our ability to establish causality and evaluate outcomes. However, despite being a secondary analysis of an existing database, with the available data it was possible to extensively calculate cardiovascular risk predictive scores, highlighting the higher cardiometabolic risk in DO compared to other anthropometric and muscle strength profiles. Also, because of the several covariates used for risk score calculation, we opted for means comparison of groups instead of adjusted regression models, however, the comparisons that exhibited significant results presented elevated statistical power (IA = 0.94; HDL-c = 0.95; Catelli I = 0.95; and Castelli II = 0.95). Another relevant limitation of the study was the occurrence of the COVID-19 pandemic that led us to interrupt data collection during the ESAT study.

## 5. Conclusions

The results of the present study suggest that individuals with DO exhibit elevated cardiometabolic risk, mainly related to atherosclerosis risk, compared to other anthropometrical and peripheral muscle strength profiles. Although our results highlight the importance of the need for diagnosis and monitoring of DO in the context of cardiovascular disease prevention, prospective and interventional studies are needed to confirm such observations and better understand the underlying mechanisms of DO and cardiovascular risk. Nevertheless, our findings emphasize the need to create primary prevention policies with possibility of the incorporation of muscle strength assessment into the primary care routine, as well as promoting the need to develop rehabilitation and prevention programs, with a focus on precision and personalized medicine.

## Figures and Tables

**Table 1:** Formulas for predictive cardiovascular risk indices.

CARDIOVASCULAR RISK INDEX/INDICATOR	FORMULA	PREDICTIVE RISK VALUES
**Atherogenic Index (AI)**	$AI=\frac{TC-HDL}{HDL}$	Low cardiovascular risk (<2)
**Plasma Atherogenic Index (PAI)**	$PAI=\log\left(\frac{TG}{HDL}-c\right)$	Low (<0.11) Medium (0.11–0.21) High (>0.21)
**Hypertriglyceridemic Waist (HW)**	-	WC ≥ 88 cm (women) ≥102 cm (men) and TG ≥ 150 mg/dL (both sexes)
**A Body Shape Index (ABSI)**	$ABSI=\frac{WC}{{BMI}^{\frac{2}{3}}\times {Height}^{\frac{1}{2}}}$	-
**Atherogenic Dyslipidemia (AD)**	-	TG ≥ 150, HDL-c < 40 and LDL-c > 130 (men) TG ≥ 150, HDL-c < 50 and LDL-c > 130 (women) Or TG ≥ 150, HDL-c < 40 and LDL-c > 130 (both sexes)
**Castelli’s Index I (CI I)**	$CII=\frac{TC}{HDL-c}$	IC I > 4.4 (women), >5.1 (men);
**Castelli’s Index II (CI II)**	$CIII=\frac{LDL-c}{HDL-c}$	IC II > 2.9 (women), >3.3 (men)
**Framingham Score**	-	Low (<5%), Moderate (5–19%), High (≥20%)

TC = Total Cholesterol; HDL-c = High-Density Lipoprotein Cholesterol; TG = Triglycerides; WC = Waist Circumference; LDL-c = Low-Density Lipoprotein Cholesterol.

**Table 2:** Descriptive data of study participants.

Variable	*n* = 199 Mean ± SD or Median [IQR 25–75%] or N (%)
**Age (years)**	45.1 ± 11.7
**Sex (%)**	
Men	81 (40.7%)
Women	118 (59.3%)
**Weight (Kg)**	76.8 [68.2–88.9]
**Height (m)**	1.65 [1.58–1.73]
**BMI (Kg/m^2^)**	28.2 [24.8–32.1]
**BMI categories**	
Normal weight	52 (26.1%)
Overweight	73 (36.7%)
Obese	74 (37.2%)
**Waist circumference (cm)**	92 [83–101]
**Handgrip strength (KgF)**	28 [22–40]
Men	40 [34–47]
Women	23 [20–28]
**Dynapenia**	91 (45.7%)
**IPAQ-SF categories**	
Low	64 (32.2%)
Moderate	59 (29.7%)
High	76 (38.2%)
**Comorbidities**	
Hypertension	41 (20.6%)
Diabetes	11 (5.5%)
Dyslipidemia	3 (1.5%)
Hypothyroidism	9 (4.5%)
Smoking	
*Non-smoker*	147 (73.9%)
*Smoker*	20 (10.0%)
*Former smoker*	32 (16.1%)
**Blood Glucose (mg/dL)**	89 [84–97]
**Total Cholesterol (mg/dL)**	185 [160–212]
**HDL Cholesterol (mg/dL)**	50 [42–60]
**LDL Cholesterol (mg/dL)**	127 [102–153]
**Triglycerides (mg/dL)**	102 [74–147]
**Medications**	
Antihypertensive Medications	35 (17.6%)
Diuretics	13 (6.5%)
Antidiabetic Medications	10 (5%)
Antihyperlipidemic Medications	7 (3.5%)
Psychotropic Medications	14 (7%)
**Cardiometabolic risk assessment**	
Atherogenic Index	2.7 [2.0–3.4]
Plasma Atherogenic Index	0.29 [0.12–0.51]
Plasma Atherogenic Index	
*Low Risk*	45 (22.6%)
*Intermediate Risk*	30 (15.1%)
*Increased Risk*	124 (62.3%)
Hypertriglyceridemic Waist	26 (13.1%)
A Body Shape Index	0.77 [0.73–0.80]
Atherogenic Dyslipidemia	11 (5.53%)
Castelli I	3.7 [3.0–4.4]
Castelli I risk	48 (24.1%)
Castelli II	2.5 [2.0–3.3]
Castelli II risk	74 (37.2%)
Framingham (%)	5.6 [3.3–11.7]
Framingham risk category	
*Low risk (<5%)*	87 (43.7%)
*Intermediate risk (5–19%)*	87 (43.7%)
*High risk (≥20%)*	25 (12.6%)

BMI = Body Mass Index, IPAQ-SF = International Physical Activity Questionnaire-Short Version, HDL = High-density Lipoprotein cholesterol, LDL = Low-density Lipoprotein cholesterol.

**Table 3:** Comparison of clinical and cardiovascular parameters among anthropometric and muscle strength profiles.

Variable	NOND (N = 68)	NOD (N = 57)	OND (N = 40)	DO (N = 34)	*p*-Value
**Age (years)**	46.7 ± 12.6	43.3 ± 11.8	48 ± 11.5	41.5 ± 8.2	0.058
**Sex (%)**					0.508
Men	30 (44.1%)	25 (43.9%)	16 (40%)	10 (29.4%)	
Women	38 (55.9%)	32 (56.1%)	24 (60%)	24 (70.6%)	
**Weight (Kg)**	71.9 [65–80]	70.9 [63–74.4]	91.4 [83.9–104.9] ***^†††^	89 [78.3–104] ***^†††^	**<0.001**
**Height (m)**	1.7 [1.6–1.7]	1.7 [1.6–1.7]	1.7 [1.6–1.8]	1.6 [1.6–1.7]	0.110
**BMI (Kg/m^2^)**	25.8 [23.8–28]	26 [23.3–27.3]	32.9 [31.5–34.7] ***^†††^	33.3 [31–37.5] ***^†††^	**<0.001**
**BMI categories**					**<0.001**
Eutrophic	27 (39.7%)	25 (43.9%)	0	0	
Overweight	41 (60.3%)	32 (56.1%)	0	0	
Obese	0	0	40 (100%) ***^†††^	34 (100%) ***^†††^	
**Waist circumference (cm)**	85 [79–93]	85 [80–92]	103.1 [95–112] ***^†††^	103.3 [96.4–110] ***^†††^	**<0.001**
**Handgrip strength (Kg/F)**	38 [26–45]	22 [19–32] ***	32 [27–49] **^†††^**	22 [20–28] ***^###^	**<0.001**
Men	44 [40–50]	34 [30–36] ***	49 [42–53] **^†††^**	35 [29–36] ***^###^	**<0.001**
Women	27 [26–30]	20 [18–22] ***	28 [25–31>] **^†††^**	21 [19–22] ***^###^	**<0.001**
**Dynapenia**	0 (0%)	57 (100%)	0 (0%)	34 (100%)	**<0.001**
**IPAQ-SF categories**					0.650
Low	16 (23.5%)	19 (33.3%)	16 (40%)	13 (38.2%)	
Moderate	22 (32.4%)	17 (29.8%)	11 (27.5%)	9 (26.5%)	
High	30 (44.1%)	21 (36.9%)	13 (32.5%)	12 (35.3%)	
**Comorbidities**					
Hypertension	10 (14.7%)	10 (17.5%)	11 (27.5%)	10 (29.4%)	0.200
Diabetes	4 (5.9%)	3 (5.3%)	3 (7.5%)	1 (2.9%)	0.880
Dyslipidemia	0 (0.0%)	0 (0.0%)	3 (7.5%)	0 (0.0%)	**0.012**
Hypothyroidism	2 (2.9%)	4 (7.0%)	1 (2.5%)	2 (5.9%)	0.666
Smoking					0.701
*Non-smoker*	53 (77.9%)	44 (77.2%)	26 (65.0%)	24 (70.6%)	
*Smoker*	6 (8.8%)	6 (10.5%)	4 (10.0%)	4 (11.8%)	
*Former smoker*	9 (13.2%)	7 (12.3%)	10 (25.0%)	6 (17.6%)	
**Glycemia (mg/dL)**	90 [84–97]	88 [83–94]	93 [87–101.5]	90 [85–99]	0.099
**Total Cholesterol (mg/dL)**	180 160–220]	182 [162–205]	191 [158–228]	188 [168–206]	0.664
**HDL Cholesterol (mg/dL)**	51 [43–62]	50 [47–66]	51 [42–60]	46 [35–52] **^†††#^	**0.009**
**LDL Cholesterol (mg/dL)**	126 [100–156]	118 [102.6]	135 [100–161]	132 [103–152]	0.751
**Triglycerides (mg/dL)**	96 [69–126]	85 [65–138]	144 [80–189] **^†††^	106 [90–139] *^††^	**0.002**
**Medications**					
Antihypertensive drugs	10 (14.7%)	8 (14%)	10 (25%)	7 (20.6%)	0.446
Diuretics	2 (2.9%)	1 (1.7%)	6 (15%) **^††^	4 (11.8%) *^†^	**0.018**
Antidiabetic drugs	2 (2.9%)	3 (5.3%)	3 (7.5%)	2 (5.9%)	0.665
Antilipemic drugs	2 (2.9%)	2 (3.5%)	3 (7.5%)	0	0.377
Psychotropic drugs	2 (2.9%)	7 (12.3%%)	2 (5%)	2 (8.8%)	0.207
**Cardiometabolic risk**					
Atherogenic Index	2.6 [2.0–3.3]	2.3 [1.6–4.1]	2.9 [2.1–3.5] **^†^**	3.1 [2.6–3.9] **^†††#^	**0.004**
Atherogenic Index of Plasma	0.2 [0.1–0.5]	0.2 [0.0–0.4]	0.4 [0.2–0.6] *^††^	0.4 [0.3–0.5] **^†††^	**0.001**
Atherogenic Index of Plasma categories					**0.002**
*Low Risk*	17 (25%)	21 (36.8%)	6 (15%)	1 (2.9%)	
*Intermediate Risk*	13 (19.1%)	8 (14%)	4 (10%)	5 (14.7%)	
*High Risk*	38 (55.9%)	28 (49.1%)	30 (75%) *^††^	28 (82.4%) **^†††^	
Hypertriglyceridemic Waist	2 (2.9%)	2 (3.5%)	16 (40%) ***^†††^	6 (17.7%) *^†##^	**<0.001**
A Body Shape Index	0.8 [0.7–0.8]	0.8 [0.7–0.8]	0.8 [0.7–0.8]	0.8 [0.7–0.8]	0.806
Atherogenic Dyslipidemia	4 (5.9%)	2 (3.5%)	4 (10%)	1 (2.9%)	0.530
Castelli I	3.5 [3.0–4.3]	3.3 [2.6–5.1]	3.8 [3.1–4.5] **^†^**	4.1 [3.6–4.9] **^†††#^	**0.005**
*Castelli I risk*	14 (20.6%)	13 (22.8%)	10 (25%)	11 (32.35%)	0.608
Castelli II	2.5 [2.0–3.2]	2.4 [1.6–3.0]	2.6 [2.0–3.3]	2.9 [2.5–3.5] **^††#^	**0.020**
*Castelli II risk*	24 (35.3%)	18 (31.6%)	14 (35%)	18 (52.9%)	0.219
Framingham (%)	5.7 [2.5–13.8]	4.3 [1.9–9.0]	7.6 [4.1–12.8]	5.7 [2.4–2.3]	0.562
Framingham risk category					0.141
*Low risk (<5%)*	31 (45.6%)	29 (50.9%)	12 (30%)	15 (44.1%)	
*Intermediate risk (5–19%)*	24 (35.3%)	23 (40.3%)	23 (57.5%)	17 (50%)	
*High risk (≥20%)*	13 (19.1%)	5 (8.8%)	5 (12.5%)	2 (5.9%)	

Data described as Mean ± SD or Median [IQR 25–75%] or N (%); NOND = Non-obese/Non-dynapenic. NOD = Non-obese/Dynapenic. OND = Obese/Dynapenic. OD = Obese Dynapenic. BMI = Body Mass Index. IPAQ-SF = International Physical Activity Questionnaire—Short Version. HDL = High-density lipoprotein; LDL = Low-density lipoprotein. Analysis performed with T-test (for age variable); Kruskal–Wallis and Fisher’s exact test, followed by post hoc Dunn’s test. vs. NOND: * *p* < 0.05; ** *p* < 0.01; *** *p* < 0.001; vs. NOD: ^†^ *p* < 0.05; ^††^ *p* < 0.01; ^†††^ *p* < 0.001; vs. OND; ^#^ *p*< 0.05; ^##^ *p*< 0.01; ^###^ *p* < 0.001.

## Data Availability

The crude data can be accessed in https://osf.io/gcvu9/ after article acceptance.
