# Correction: Carvalho et al. Increased Cardiometabolic Risk in Dynapenic Obesity: Results from the Study of Workers’ Health (ESAT). *Life* 2024, *14*, 1174

**DOI:** 10.3390/life15060906

**Published:** 2025-06-04

**Authors:** Mariana de Oliveira Carvalho, Alice Pereira Duque, Grazielle Vilas Bôas Huguenin, Mauro Felippe Felix Mediano, Luiz Fernando Rodrigues Júnior

**Affiliations:** 1 Education and Research Department, National Institute of Cardiology, Rio de Janeiro 22240-002, RJ, Brazil; mariana.olicarvalho@gmail.com (M.d.O.C.); alicepereiraduque@gmail.com (A.P.D.); graziellevbhuguenin@gmail.com (G.V.B.H.); mffmediano@gmail.com (M.F.F.M.); 2 Nutrition and Dietetics Department, Fluminense Federal University, Niterói 24020-140, RJ, Brazil; 3 Evandro Chagas National Institute of Infectious Diseases, Oswaldo Cruz Foundation, Rio de Janeiro 21040-900, RJ, Brazil; 4 Department of Physiological Sciences, Federal University of the State of Rio de Janeiro, Rio de Janeiro 20211-010, RJ, Brazil

In the original publication, there was a mistake in Table 3. Comparison of clinical and cardiovascular parameters among anthropometric and muscle strength profiles [1]. The values describing triglycerides are published as follows:


**Variable**

**NOND**

**(N = 68)**

**NOD**

**(N = 57)**

**OND**

**(N = 40)**

**DO**

**(N = 34)**

***p*-Value**

**Triglycerides (mg/dL)**
96 [69–126]85 [65–189] **^†††^106 [90–139] *^††^
**0.002**


The corrected information regarding triglycerides in Table 3. Comparison of clinical and cardiovascular parameters among anthropometric and muscle strength profiles appears below.


**Variable**

**NOND**

**(N = 68)**

**NOD**

**(N = 57)**

**OND**

**(N = 40)**

**DO**

**(N = 34)**

***p*-Value**

**Triglycerides (mg/dL)**
96 [69–126]85 [65–138]144 [80–189] **^†††^106 [90–139] *^††^
**0.002**


The authors state that the scientific conclusions are unaffected. This correction was approved by the Academic Editor. The original publication has also been updated.
